# A Study on the Uncertainty of a Laser Triangulator Considering System Covariances

**DOI:** 10.3390/s20061630

**Published:** 2020-03-14

**Authors:** Pablo Puerto, Beñat Estala, Alberto Mendikute

**Affiliations:** IDEKO, Elgoibar, Guipuzcoa 20870, Spain; bestala@ideko.es (B.E.); amendikute@ideko.es (A.M.)

**Keywords:** laser triangulation, accuracy, covariances, uncertainty, camera calibration, laser calibration

## Abstract

A laser triangulation system, which is composed of a camera and a laser, calculates distances between objects intersected by the laser plane. Even though there are commercial triangulation systems, developing a new system allows the design to be adapted to the needs, in addition to allowing dimensions or processing times to be optimized; however the disadvantage is that the real accuracy is not known. The aim of the research is to identify and discuss the relevance of the most significant error sources in laser triangulator systems, predicting their error contribution to the final joint measurement accuracy. Two main phases are considered in this study, namely the calibration and measurement processes. The main error sources are identified and characterized throughout both phases, and a synthetic error propagation methodology is proposed to study the measurement accuracy. As a novelty in uncertainty analysis, the present approach encompasses the covariances of correlated system variables, characterizing both phases for a laser triangulator. An experimental methodology is adopted to evaluate the measurement accuracy in a laser triangulator, comparing it with the values obtained with the synthetic error propagation methodology. The relevance of each error source is discussed, as well as the accuracy of the error propagation. A linearity value of 40 µm and maximum error of 0.6 mm are observed for a 100 mm measuring range, with the camera calibration phase being the main error contributor.

## 1. Introduction

To guarantee accurate measurement using a triangulation system, previous calibration procedures must be performed in order to know the relative position between both elements. These measurement systems are also known as laser displacement sensors (LDS), structured-light sensors, or sheets of light. The calibration process is carried out with a reference object with previously known dimensions. This object can either be 2D or 3D [1]. The 2D objects are planes containing markers or lines to detect their position [2], while 3D objects facilitate positioning [3]. Normally, more than the position of the object is needed unless the 3D object is accurate enough to define the laser plane.

According to the bibliography, as mentioned previously, calibration of a triangulation system consists of projecting a laser line onto a calibration rig; if the laser line is projected onto more than a single plane (3D object), this adds accuracy to the result [4,5]. This also requires prior camera calibration, so that the laser line is detected correctly in the image [6].

Therefore, the triangulation accuracy depends on the calibration methods, as well as the calibration patterns used [7,8]. The bibliography shows that calibration procedures are distinguished by both 2D and 3D pattern objects. Even if a 2D pattern is used in laser calibration, it is possible to use a 3D object to measure plane heights [9] and verify the previous calibration [7]. Furthermore, in photogrammetry, other methods such as Monte Carlo analysis are used in order to compare the results taken from the tests with the synthetic ones, contrasting accuracy, standard deviations, and repeatability between calibrations [8,10]. This method consists of the propagation of uncertainty, which solves system uncertainties that are not able to be determined analytically. The Monte Carlo method is used in order to obtain distortion values for a specific calibration, depending on the distortion values of the variables (extrinsic values, image detection, marker’s 3D positioning) and the number of iterations. The main idea is to adjust this approximated uncertainty in order to obtain similar errors and covariances to the real ones [11].

## 2. Measurement Principle and Uncertainty Analysis Methodology

In order to predict the accuracy of the laser triangulation system, an explanation of the mathematics used is shown.

### 2.1. Triangulation System

As mentioned, a laser triangulation system consists of a camera and a laser line. The first step is the detection of the line using a peak detector algorithm. The mass center is calculated using the column. This research is not focused on the optimization of the detection, so the test avoids the influence of tilt signals. However, the lens is not perfect and it must be corrected using the detection points. There are several models to represent this effect, but the most widespread is Brown’s model, which corrects the barrel, pin cushion, and mustache distortions. In order to calculate theses parameters, also known as intrinsic parameters, a calibration process is carried out, which is explained in Section 3.3.1. Once the center of the line is detected and corrected, the pixels are converted into distances using the features of the camera. The camera is modelized following the perspective projection principle, taking into account the focal distance and the size of the pixels, which are also calculated in the camera calibration. To determine the position in 3D, the relative position between the camera and laser must be considered. The plane of the laser and the line from the detected pixel to the focal point crosses in a 3D point, giving the spatial coordinate. In order to determine the relative position between a camera and a laser plane, the second calibration process is carried out (see Section 3.3.2).

**Figure 1:**
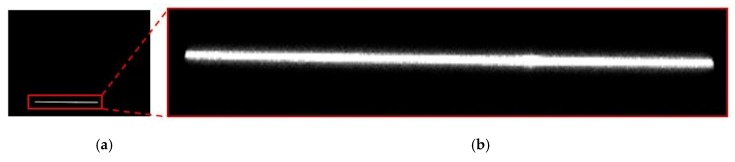
Laser line displayed horizontally in the image: (**a**) complete image taken by the camera; (**b**) laser line section obtained from the complete image.

In order to detect the laser line in the image (see Figure 1), an internal software is used, which consists of taking each vertical pixel column and getting each pixel’s light intensity (0–255). With this information, the software only considers pixels with an intensity value over 10, and by applying a normal distribution to those pixels, the pixel in which the normal distribution maximum value is positioned will be considered as the laser line center. Following the criteria mentioned earlier, an average value of 10.4878 pixels was obtained for laser width in Y camera axis.

**Figure 2:**
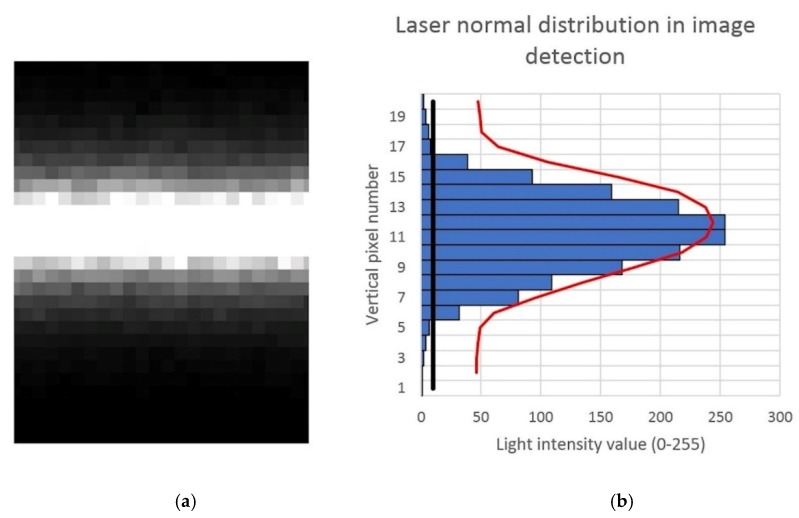
Laser line detection procedure in image: (**a**) part of the laser line detection for each pixel; (**b**) pertinent histogram for a pixel column with each pixel’s light intensity value.

As can be seen in Figure 2, part of a vertical pixel column was graphed, and considering only pixels with a light intensity value over 10, the maximum value of its normal distribution was obtained in pixel number 11.347, with this pixel considered the laser line center.

### 2.2. Monte Carlo Method

The uncertainty results [12] of 3D points obtained by measurement with laser triangulation is linked with the uncertainty of the inputs. According to GUM (Guides to the expression of Uncertainty in Measurement) [13], uncertainty is determined by the non-negative parameter, which represents the dispersion of a value for a measurement and its precision parameter. This system cannot be used to solve problems analytically because the intrinsic parameters are solved by a non-lineal equation. In these cases, it is recommended to use the Monte Carlo method, which consists of generating a vector, with the deviation representing the experimental observations as an input. According to GUM, the input variables must be determined by probability distributions, considering their normal distribution.

This Monte Carlo analysis is applied in intrinsic parameters, image detection, and positioning in space (probing with CMM (Coordinate Measuring Machine)). The obtained values of these factors depend on the conditions where calibrations are carried out.

Moreover, the number of iterations for Monte Carlo analysis is also relevant for the final result. Within a Monte Carlo simulation, the computational steps are repeated between 1000 and 1,000,000 times [13], using a value of 1000 iterations.

### 2.3. Covariance Among the Parameters

In this research, one of the main novelties is the consideration of the covariance of the parameters to increase the prediction of the system. This system is appropriate for use because the covariance takes part in three steps.

In the first phase, the uncertainty of the CMM must be considered to generate the spatial position of the planes. After this, the covariance of the output is checked to compare it with the experimental results. The uncertainty values of the laser parameters are dependent on each other because they are calculated at the same time. With this information, in the final step, the inputs of the relative positions of the camera and laser must be generated using covariance values.

The laser parameters mentioned earlier define the laser plane position and orientation, which are the normal vector of the laser plane $n_{L}$ and the Z coordinate where the laser intersects the Z-axis in the camera coordinate system, $l_{L}$, which always has the components $\left[0\;0\;l_{L}\right]$.

In order to generate the inputs for the algorithms, Guidelines for the Expression of Uncertainty in Measurement [13] have been used, following the expression below:

(1) $$s\left(y_{i},z_{i}\right)=\frac{1}{n-1}\sum_{j=1}^{n}\left(y_{j}-\bar{y}\right)\left(z_{j}-\bar{z}\right)$$

where $y_{j}$ and $z_{j}$ are individual observations of the quantities y and z, while $\bar{y}$ and $\bar{z}$ are calculated by the arithmetic mean of each component, obtaining the covariance between $y_{i}$ and $z_{i}$ as a result.

This expression offers the correlation value between two components, which is associated with a covariance between them. By applying this equation to all the combinations between components in the laser calibration ($n_{L}\left[0\;0\;n_{L_{Z}}\right]$ + $l_{L}$), a covariance matrix is obtained, as shown in Section 5.1.

### 2.4. Measurement by Triangulation

**Figure 3:**
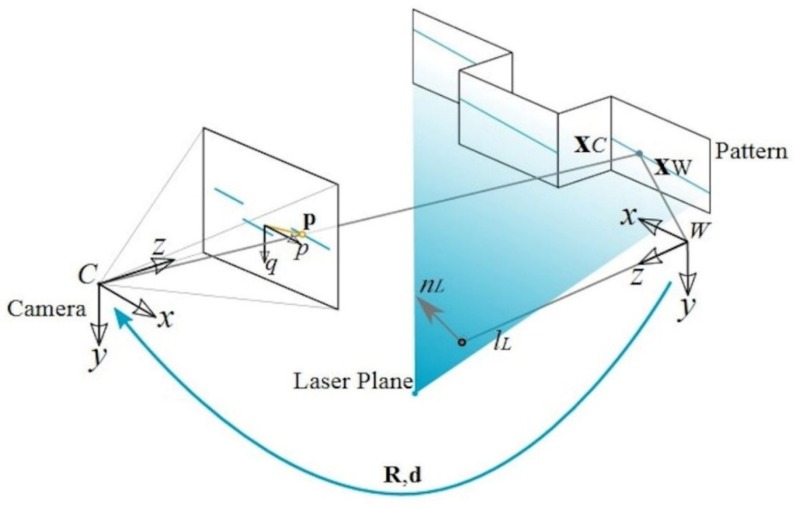
Laser triangulation system. The calibration processes consist of identifying extrinsic and intrinsic parameters of both elements followed by the measurement process for the image. Here, $x_{C}$ and $x_{W}$ correspond to laser point p taken in the image and projected in the pattern following unitary vector u, for both the camera coordinates (C) and world coordinates (W), with a transformation matrix R,d. Additionally, $n_{L}$ and $l_{L}$ correspond to laser parameters.

The measurement process for laser triangulation consists of locating a three-dimensional point $x_{w}$ starting from a retroprojection of a point in image p. As can be observed in Figure 3, an infinite number of points can be projected in p, where the unitary vector u corresponds to the direction from camera coordinates to p. However, if those points are restricted to the laser plane characterized by $n_{L}$ and $l_{L}$, only a single point exists that corresponds to the intersection of the direction of vector u and the laser plane.

(2) $$x_{C}=\lambda u$$

Firstly, point p has to be defined in the image, which gives its three-dimensional intersection point with the laser plane following the u vector, giving us $x_{C}$ and $x_{W}$ for the camera system and world system, respectively:

(3) $$x_{C}=Rx_{W}+d$$

We remove $x_{W}$ from the previous equation:

(4) $$x_{W}=R^{T}x_{C}-R^{T}d$$

We replace Equation (2) in Equation (4):

(5) $$x_{W}=\lambda R^{T}u-R^{T}d$$

Moreover, it can be demonstrated that points that intersect the laser plane follow as below:

(6) $$n_{L}\cdot x_{W}=\rho L$$

where $\rho L=n_{L}l_{L}$. Replacing Equation (5) in Equation (6) gives us:

(7) $$n_{L}\cdot \left(\lambda R^{T}u-R^{T}d\right)=\rho L$$

Finally, removing λ, we obtain the following equation:

(8) $$\lambda =\frac{\rho L+n_{L}\cdot \left(R^{T}d\right)}{n_{L}\cdot \left(R^{T}u\right)}$$

Once λ is obtained, $x_{W}$ can also be determined by replacing Equation (8) with Equation (5).

### 2.5. Flow Chart

**Figure 4:**
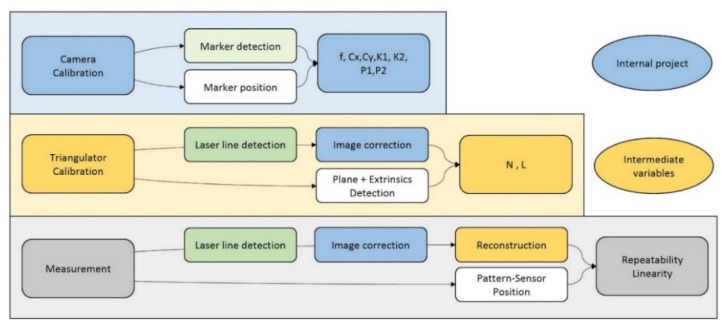
Flow chart for experiments, with each block divided into different phases. Each output obtained in camera and laser calibrations is used as an input for block measurements. For camera calibration, the final outputs are the intrinsic parameters, which are the focal length (f), centre of distortions (Cx, Cy), and calibration parameters (K1, K2, P1, P2). Both N and L are triangulator calibration parameters defining the laser position in space.

As explained at the beginning of this paper, the experimental analysis is divided into three different blocks (see Figure 4): camera calibration, triangulation system calibration, and measurement. Moreover, each of these blocks has sub-phases, such as detection, correction, or positioning, where the output of the previous phase (camera calibration) takes part in other phases (triangulator calibration) as a new input [14].

Firstly, a correct camera calibration is essential for both triangulator calibration and measurements, having an important role in the system. This calibration consists of taking images and detecting a marker placed in a coordinate measurement machine’s stylus in different positions. These detected positions are compared with the marker position provided by the CMM, obtaining the camera’s intrinsic parameters. More details of this process are provided in [8].

The second step is the triangulator calibration, which consists of casting the laser upon an object and detecting the laser line in the image. This image is corrected by the intrinsic parameters obtained in camera calibration, after which the object plane and laser extrinsic parameters are detected, finally obtaining the $n_{L}$ and $l_{L}$ parameters from the laser. This calibration is also carried out in the CMM, providing the position of the object.

Finally, the measurement phase consists of casting the laser upon ceramic blocks with specific heights while detecting the laser line and correcting the image. With the information for the laser and block positions and the triangulator variables, it is possible to calculate the height, repeating this process to obtain the system’s repeatability and linearity.

## 3. Experiments and Triangulator Characterization

The following step is to define all the experiments and measurements to be performed with the laser triangulator. In order to simulate laser triangulation behaviour, it is essential to analyze the variation of the inputs, such as camera calibration, laser calibration, and height measurement. Depending on the way these tests are carried out, the accuracy can be highly variable, so one of the main objectives is to find out the influence of the input’s variation in the final measurement.

### 3.1. Elements Summary

In order to understand all the elements taking part in the experiments, a brief description of each one follows below.

One of the main elements is the camera. In this case, a Genie HM1024 camera is used, which has a resolution of 1024 × 768 pixels, with each pixel measuring 7.4 µm. Regarding the frame rate, the camera works at 117 fps, a value which is sufficient for our experiments. Completing the triangulation system, a Prophotonix 300-0828-00 laser is used for the tests, with an opening angle of 45° and a focus distance of 300 mm, having a range of +/- 150 mm from the nominal value. To ensure a perpendicular angle between the laser and the main base for the gauge block measurements, a 6-degree Thorlabs stage is used, meaning it is possible to fix the rotations properly. This element also allows specific variations to be created in all rotations, which enables analysis of the influence of rotations in gauge block measurements. The actuators can achieve an accuracy of 5.0 µm. For the height measurements, Mitutoyo grade 0 ceramic gauge blocks were selected for measurement, since they have low uncertainty in height. The gauge blocks used for the tests are listed in Table 1, along with their nominal and deviation values, using multiples of 10 mm, ranging from 10 to 100 mm.

Although the deviation and uncertainty in ceramic blocks are provided in the table above, they are considered irrelevant because the values are under 0.25 µm, whereas the results should be around 20–50 µm.

**Figure 5:**
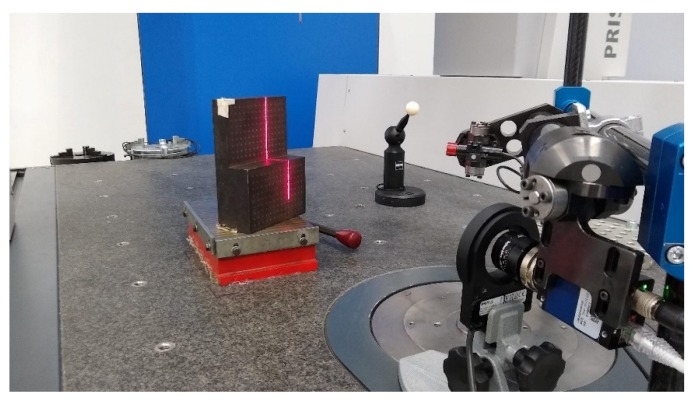
Laser calibration in the coordinate measurement machine (CMM) and the three-dimensional (3D) pattern for laser line detection. The 3D pattern position is obtained by the CMM stylus and the laser is calibrated by displaying the laser line onto its surface while the camera captures images.

On the other hand, a coordinate measurement machine (CMM) is used for calibration methods, for both the camera and triangulator calibrations. This Prismo-Zeiss machine has an accuracy of 0.9 + L/350μm. In this case, considering the working range (300 mm in Y), the overall accuracy is 2 μm [15]. For the triangulator calibration, a pattern is also needed, which is a 3D object with two different parallel planes (see Figure 5). Those planes have a flatness of 10 µm, with a difference of 35 mm in height between them. This object allows the laser triangulator to reflect the laser plane in both heights, displaying a laser line in the image for each pattern plane.

### 3.2. Triangulation System Setup

**Figure 6:**
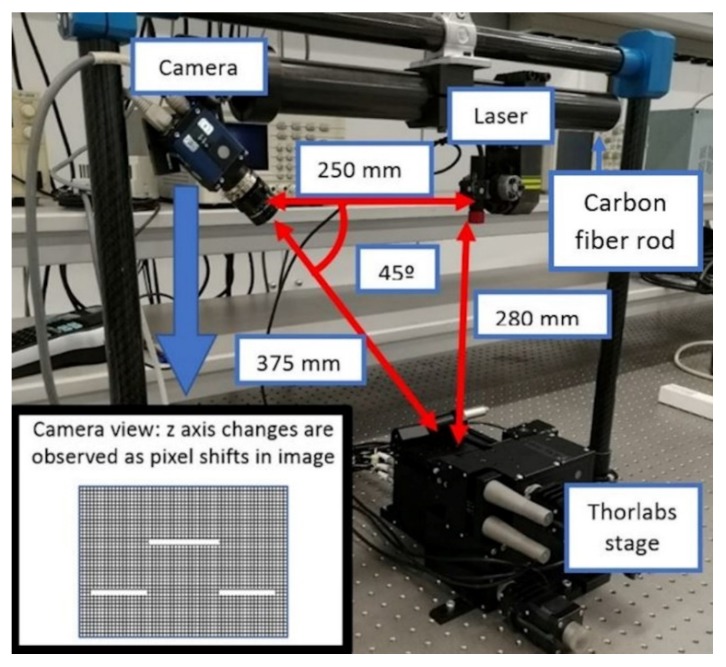
Main layout for experiments, including a camera and laser with a carbon fiber rod, along with the Thorlabs stage. At the bottom of the image, a block measurement is displayed in the camera.

To perform the experiments mentioned above, a non-commercial triangulation system was built. For this, the layout design is shown in Figure 6. The main criteria for the design are the measurement range of the laser and the focus range of the camera.

Apart from this, as shown in Figure 6, the chosen angle between the camera and laser is 45 degrees. The main reason to use this orientation is that it is the optimal position, where the light reflected by the object and the triangulation angle are compensated by themselves.

The camera and laser are clamped on a carbon fiber rod, so that their relative positions remain fixed and constant during all experiments.

### 3.3. Experiments Summary

After describing the main elements of the experiments, the next step is to determine the tests to be performed. Table 2 provides a brief description for each test, indicating the main objective and the expected outputs for them.

It is a remarkable fact that the laboratory in which these experiments were performed has a controlled temperature of 20 °C, which is constantly measured, with a maximum acceptable temperature variation of ± 0.5 °C. Moreover, the clamp that joins the camera with the laser is made from carbon fiber to avoid thermal deformations, while the ceramic blocks used for measurement have a low coefficient of expansion due to the material.

#### 3.3.1. Camera Calibration in CMM

**Figure 7:**
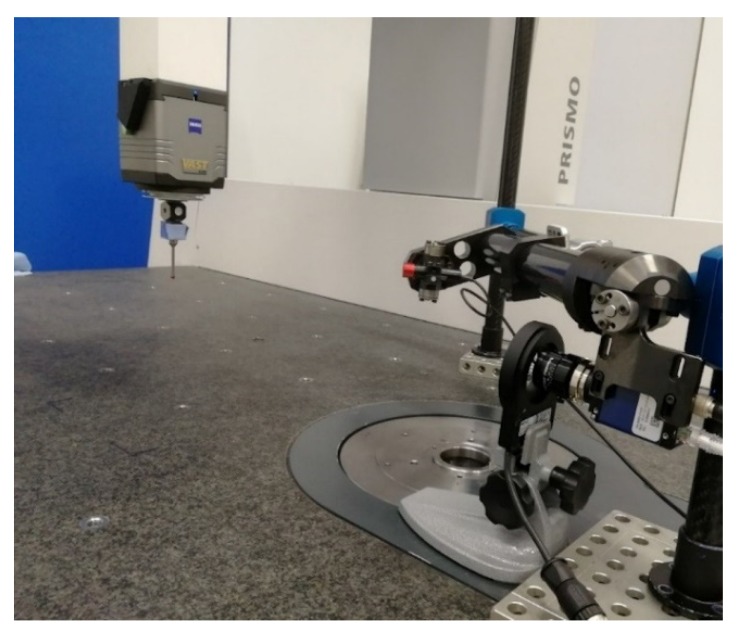
Triangulation system in the CMM and camera calibration with a marker in the CMM stylus. The marker goes through different positions, while the camera obtains images for each position in space.

**Figure 8:**
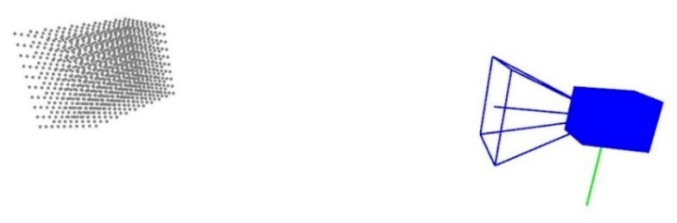
A virtual pyramid for camera calibration with 1000 points (10 × 10 × 10), which is displayed in an internal software to obtain intrinsic and extrinsic parameters.

For the camera calibration, the CMM generates a virtual pyramid (see Figure 7 and Figure 8), and by positioning a retroreflective marker on the stylus system, the CMM places the marker on those points in space while the camera takes images for each position, simulating a 3D object with numerous markers that need to be detected. After this, the images are processed by detecting the center of the marker, and once these images are matched with each position taken from the CMM, the camera´s intrinsic and extrinsic parameters are obtained. As mentioned before, this process is explained in [8].

#### 3.3.2. Laser Calibration in CMM

In the same way, the laser calibration method is also carried out inside the CMM. For this a pattern is also used, which has two heights. The stylus probes numerous points on each plane, obtaining those coordinates and adjusting them into two different planes, along with their normal vectors.

Once this is done, the next step is to display the laser in both planes (see Figure 5) while the camera acquires an image of the laser line on the pattern. The main purpose of the image is to detect the laser points on the image and match those points with the 10 planes generated by the CMM information. In doing so we can identify the laser plane’s parameters, which are the normal vector of the laser plane $n_{L}$ and the distance where the laser intersects the Z-axis in the camera coordinate system $l_{L}$, which always contains components $\left[0\;0\;l_{L}\right]$.

#### 3.3.3. Gauge Block Measurement

**Figure 9:**
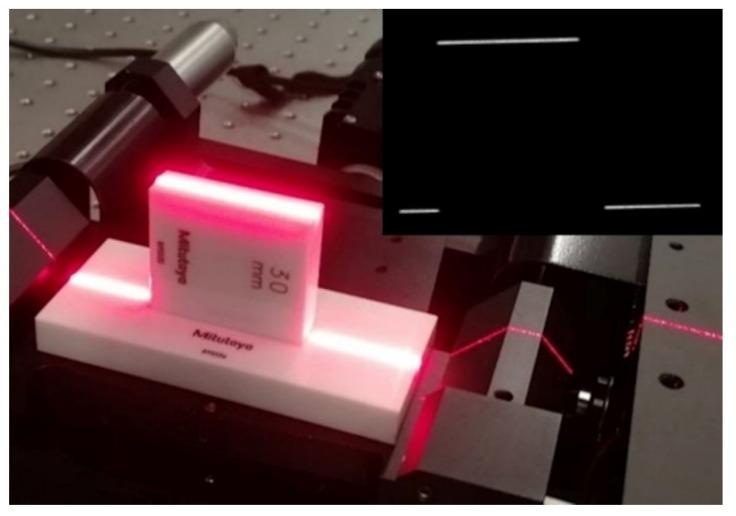
Gauge block measurement on Thorlabs stage to ensure alignment. Another block is placed to ensure a flat and reflective surface (such as the 30 mm block).

Finally, after performing all calibration tests, triangulation measurements were made (see Figure 9). The main reason for these measurements is to analyze the accuracy of the system. In this way, the chain of uncertainty is characterized. As can be seen in Figure 9, another ceramic block is positioned at the base in order to provide a flat and reflective surface, such as the measuring block’s surface (30 mm block). The block’s surfaces are reflective, avoiding laser detection differences in the image. The laser line projected on the Thorlabs stage is not taken into consideration for height measurement; only the part which is projected in both blocks is considered.

Moreover, another issue to analyze in this research is the covariance in all the inputs mentioned previously. Regarding camera calibration covariance, both the extrinsic and intrinsic parameters are solved at the same time, so there is a covariance relationship among them. In the same way, when the laser calibration is carried out, the normal vector of the laser plane ($n_{L}$) and the distance where the plane intersects the Z-axis on the coordinate system ($l_{L}$, i.e., X:0 Y:0 Z:100 mm) are also related to each other because they are part of the minimization problem. Finally, the measurement procedure for the ceramic gauge blocks is affected by the previous covariances.

After measuring 10 gauge blocks, the results are fitted to a line and the differences with respect to this line and the absolute values are shown. These are the main features of a commercial laser measurement system.

## 4. Results

**Figure 10:**
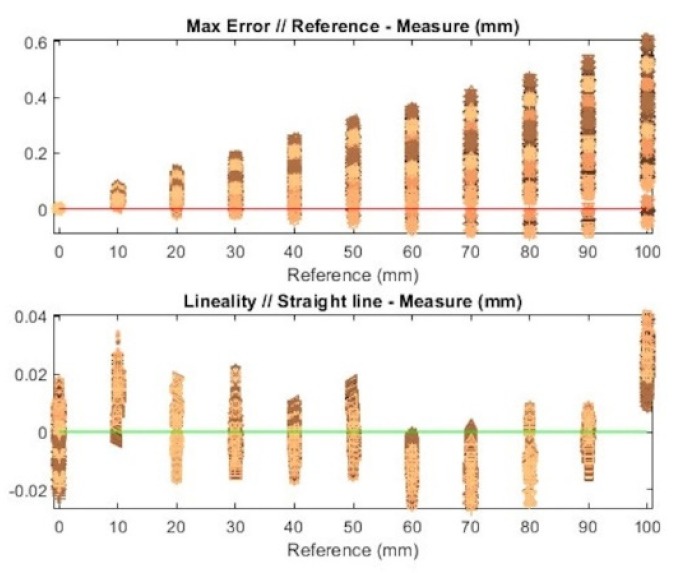
Maximum error and linearity for calibration combinations in block measurements between reference heights and obtained heights.

Once all the tests are done, the next step is to analyze those experiments and express results about measurement accuracy and the influence of different phases (camera calibration, laser calibration, and measurement) in the measurement repeatability. These results compare each ceramic block against the ideal ones (see Figure 10).

First, the maximum error of each measurement indicates the accuracy of the previous calibration methods, where this error is expected to increase directly with the block’s height. Besides this, the linearity between different heights indicates the tendency each measurement has, along with both factors for each calibration combination between the camera and laser.

As seen in the figure above, each measurement is carried out, combining all the repetitions (10 repetitions each) of the camera calibration, laser calibration, and image acquisition of the ceramic blocks, in order to obtain the influence of each of these procedures on the result.

**Figure 11:**
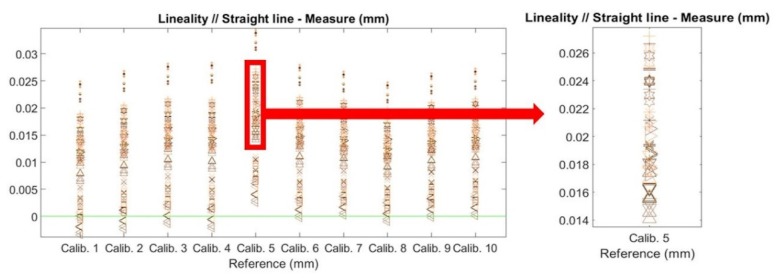
Measurement combination distribution for ten different camera calibrations, with each calibration divided into different measurements.

For each height, as seen in Figure 10, the colors represent measurements with different laser calibrations; in other words, each color (laser calibration) has a combination of 10 camera calibrations and 10 block image acquisitions (100 points). For each color, 10 different symbols exist, which represent different block measurements (10 measurements total). Finally, each colored symbol is repeated for all camera calibrations (10 calibrations total), as can be observed in Figure 11.

Since Figure 10 does not clearly offer values for the maximum error and linearity, Table 3 contains mean values and standard deviations for both the maximum error and linearity for each height. For block number 0, there is no maximum error, since it is taken as initial position, while the mean value and standard deviation increase with height, as is expected.

**Figure 12:**
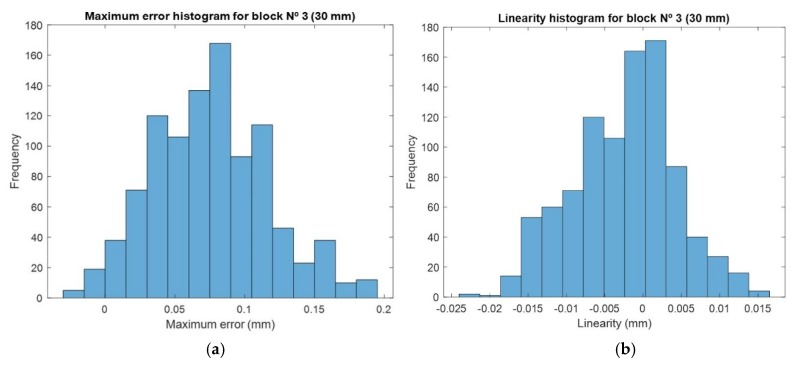
Histogram of all combinations for 30 mm height measurement: (**a**) histogram for maximum error values; (**b**) histogram for linearity values.

In addition to the table mentioned above, histograms for the maximum error and linearity are shown in Figure 12. They represent the distribution of values considering all the calibration combinations, along with the tendency for the mean values obtained for the maximum error (0.0758 mm) and linearity (−0.0025) from Table 3.

### 4.1. Maximum Error

One of the variables to discuss is the maximum error for each experiment compared with the real block height. As mentioned earlier, each height has different measurement combinations, and following the prediction of the increasing maximum error along with the height, an error value of 0.6 mm is obtained for a height measurement of 100 mm.

Additionally, it is essential to know that by considering the orientation error of the triangulation system, the camera will always give a larger height value than the actual height.

Considering the previous statement, the maximum error graphic shows that nearly all the measurements are over the nominal value, so that most of the repetitions for the measured height are higher than the real one.

Focusing on the variability with different combinations, this fact also increases with height, reaching a variability of 0.601 mm, while the mean maximum error is 0.286 mm, with a standard deviation of 0.123 mm.

### 4.2. Linearity Analysis

Following the linearity of the experiments, it is an interesting fact that the form varies along with the height (see Figure 10). Even the reference height 0 has an average linearity of 0, while small height blocks have a positive linearity, which decreases with the convex shape until reaching a value of 0 with a height of 30 mm. Then, it continues to decrease until reaching a minimum value of 60 mm, before representing the same shape the other way around.

Analyzing each height separately, in most of the blocks, the linearity is approximately 30 µm, while as with the maximum error, the mean value is 0.997 mm, giving a standard deviation of 0.00133 mm.

### 4.3. Calibration Influences in Repeatability

The next issue to be discussed is the influence of the experiments carried out in Section 3.3. Essentially, this regards the differences in results in terms of the repeatability of an experiment, regarding both the maximum error and linearity. Simply by looking at Figure 11, the most influential aspects are the camera and laser calibration, in terms of both maximum error and linearity. The influence values in percentage are shown in Table 4.

## 5. Comparison Between Experimental and Error Propagation Method

As mentioned in Section 2.2, assuming distortions with inputs, such as the 3D pattern probing error and 2D error in the image, the main objective of this section is to compare the measurement heights obtained via the experimental data with the Monte Carlo analysis. Furthermore, covariances are also considered in this chapter, comparing the experimental data with the synthetic data, exploring how related output variables are with each other. Considering the information mentioned earlier, the aim is to obtain a similar covariance matrix and measurement errors as the experimental ones obtained from the Monte Carlo analysis.

### 5.1. Calibration Comparison

Firstly, covariances from the experimental laser calibrations are obtained, which are shown in Table 5. Here, $n_{L}$ represents the normal vector of the laser plane, which has components in X, Y, and Z, while $l_{L}$ corresponds to the Z value where the laser plane intersects the Z-axis. As can be seen, the covariances between laser $n_{L}$ components are clear, obtaining an R-value for covariance between $n_{L_{1}}$ and $n_{L_{2}}$ of 0.987, a value between $n_{L_{1}}$ and $n_{L_{3}}$ of 0.986, and between $n_{L_{2}}$ and $n_{L_{3}}$ of 1.

For Monte Carlo analysis (see Table 6), after obtaining covariances with different error combinations (3D and detection error), the covariance values were obtained using a 3D error of 20 µm to calculate the positions of the planes and a detection error in the image of 0.1 pixels. These covariances have a similar tendency, where the R correlation values for Monte Carlo analysis covariances between $n_{L_{1}}$ and $n_{L_{2}}$ = 0.851, between $n_{L_{1}}$ and $n_{L_{3}}$ = 0.839, and between $n_{L_{2}}$ and $n_{L_{3}}$ = 1.

The difference between the experimental and the simulated results is the dispersion in the Monte Carlo methodology. The source of this deviation is unknown.

Additionally, the points from covariances between $n_{L}$ and other $l_{L}$ components are much more dispersed, so these correlation values do not offer much information. For Monte Carlo covariances, these points are even more dispersed, which influences the correlation values, so that these values cannot be compared due to the dispersion of the points in both cases.

R variable corresponds to correlation coefficient, which is a value between −1 and 1 that identifies if two variables change correspondently. Meanwhile, P is the probability coefficient, which indicates if the correlation value is called statistically significant (*p* < 0.05).

Following the covariance matrices for both cases, as can be seen in Table 7, both matrices have similar values, especially in $n_{L}$ component covariances, where the errors obtained previously simulate a similar covariance matrix. The covariance matrix components involving $l_{L}$ are not as accurate as the others, but considering the dispersion in samples, the values cannot be compared between both cases.

### 5.2. Comparative Measurements 

In addition to the covariance comparison, a measurement comparison was also made between experimental and synthetic data. As mentioned in the section before, Monte Carlo analysis was carried out with a detection error of 0.1 pixels, maintaining the uncertainty in Section 5.1.

The idea is to study uncertainties and compare the results with the maximum error and variability. The results of the Monte Carlo analysis show similar maximum error values in the absolute value, however the difference is that with Monte Carlo analysis these maximum errors have both positive and negative values, which are symmetric. This is why the maximum measurement error variance with a height of 100 is 0.598 mm, while the variance for the same height with Monte Carlo analysis is 0.487 mm.

**Figure 13:**
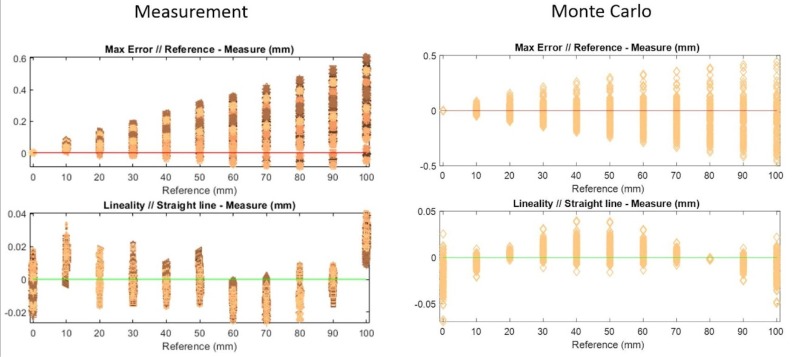
Comparative measurements between experimental and Monte Carlo analysis, with similar shapes obtained for maximum error and linearity.

For the linearity, the maximum values are similar, being slightly greater with Monte Carlo analysis. The synthetic values have similar shapes as the measured ones (see Figure 13), however in the opposite way, starting with negative linearity, increasing to a maximum positive value, and finally decreasing until they become negative values.

## 6. Paper Summary

In this paper, a laser triangulation system is designed and diverse phases are defined, such as calibration and measurement. Analyses of the maximum error and linearity for combinations of these phases repetitions are carried out and the influence of each phase (camera calibration, triangulator calibration, and measurement) is obtained in the results. In order to study the uncertainty in triangulation, covariances between laser triangulation parameters are studied, identifying main error sources and obtaining approximated values for real measurement errors using Monte Carlo analysis.

Following the mentioned analysis, a linearity of 40 µm and a maximum error of 0.6 mm is observed for a 100 mm measuring range for combinations of repetitions of different phases, while camera calibration is proven to be the main error contributor between phases. Error propagation analysis provides similar values to real ones in the covariance matrix, having such small values that the approximation is great.

## 7. Discussion

In summary, it is interesting to remark that the uncertainty study via Monte Carlo analysis is capable of recreating covariance and measurement values from experimental data, as mentioned in Section 5.1. It is also known that the most sensitive component in triangulation system characterization is the $l_{L}$ value, which is reflected in the variability of its covariance values. Thanks to this tool, it is possible to decrease the number of tests during analysis, which is a significant improvement in terms of optimization.

Additionally, as mentioned at the beginning, a non-commercial triangulation system allows the design to reflect the owner’s needs, providing flexibility and speed compared to commercial systems. In this case, it is possible to select the camera and laser which best fit the requirements, as well as the positioning between both objects.

Referring to the results obtained for accuracy and repeatability, along with experimental measurements, it is concluded that the maximum error in the 100 mm measurement is about 0.6 mm, considering all the combinations of calibrations. To decrease this value, considering the influence table (see Table 4), a denser camera calibration should be carried out, since this is the most influential factor. Moreover, the maximum linearity between measurements is about 40 µm in 100 mm, which is a great result, although this could also be decreased with more accurate camera calibration. The maximum error using Monte Carlo analysis is bigger (57 µm in 100 mm) than the experimental error, however the error is more symmetric.

## 8. Conclusions and Future Lines

Summarizing different analyses carried out in previous sections, the information obtained by different combinations of phases shows that the most significant phase is the camera calibration, which contains 57% of the entire error, followed by the triangulator calibration with 40%, and finally the measurements with 3%. The covariance analysis follows, as seen in Table 5, where the correlation between different N components is evident. Synthetic measurement also provides similar correlation values for those components, while in the covariance matrix, even if the numbers are not equal, the decimals between both tables are the same, giving a good result considering the low dimensions of these values.

On one hand, the different trends between the experimental and Monte Carlo analyses must be clarified in both cases, regarding maximum error and linearity. Perhaps the consideration of the variable uncertainty of the pixel detection could help to find the difference.

On the other hand, the setup used gives the optimal positions for the camera and laser. The influence of the relative positions should be investigated. In some cases, it may be worthwhile sacrificing the reflection of the light to increase the resolution, or vice versa.

## Figures and Tables

**Table 1:** Block characteristics, such as nominal value, deviation, and uncertainty.

Nominal L (mm)	Deviation to Nominal D (µm)	Length Variation F (µm)	Uncertainty U (µm)
10	−0.03	0.09	0.09
20	0.04	0.08	0.10
30	0.25	0.12	0.11
40	0.18	0.09	0.12
50	0.12	0.05	0.13
60	0.22	0.09	0.14
70	0.26	0.18	0.16
80	0.21	0.25	0.16
90	0.18	0.10	0.17
100	0.14	0.17	0.18

**Table 2:** Summary of experiments.

Nº Test	Objective	Results
1	Camera calibration of CMM	Extrinsic and intrinsic camera parameters
2	Laser calibration of CMM	Intrinsic laser parameters ($n_{L}$$\;\mathrm{and}\;l_{L}$)
3	Height measurement	Ceramic gauge block heights

**Table 3:** Mean value and standard deviation for both maximum error and linearity.

Nº Block	Maximum Error		Linearity	
	$\bar{X}$ (mm)	σ (mm)	$\bar{X}$ (mm)	σ (mm)
**0**	0	0	0.0012	0.0075
**1**	0.0391	0.0158	0.0131	0.0088
**2**	0.0537	0.0285	0.0016	0.0124
**3**	0.0758	0.0414	−0.0025	0.0125
**4**	0.1009	0.0555	−0.0035	0.0163
**5**	0.1316	0.0695	0.0010	0.0199
**6**	0.1462	0.0831	−0.0105	0.0226
**7**	0.1703	0.0961	−0.0125	0.0247
**8**	0.2002	0.1088	−0.0088	0.0280
**9**	0.2341	0.1197	−0.0009	0.0308
**10**	0.2842	0.1296	0.0229	0.0332

**Table 4:** Calibration and measurement influence on repeatability.

	Maximum Error Deviation		Linearity Deviation	
	mm	%	mm	%
**Camera calibration**	0.10600	56.910	0.00116	58.617
**Laser calibration**	0.07430	39.901	0.00078	39.228
**Measurement**	0.00595	3.200	0.00004	2.155

**Table 5:** Covariance (correlation and probability) values for experimental data.

Experimental	$n_{L_{1}}$		$n_{L_{2}}$		$n_{L_{3}}$		$l_{L}$	
	R	p	R	p	R	p	R	p
$n_{L_{1}}$	-	-	-	-	-	-	-	-
$n_{L_{2}}$	0.987	0	-	-	-	-	-	-
$n_{L_{3}}$	0.986	0	1	0	-	-	-	-
$l_{L}$	−0.280	0.006	−0.270	0.007	−0.270	0.007	-	-

**Table 6:** Covariance (correlation and probability) values for Monte Carlo analysis (3D: 20 µm; image: 0.1 pixel).

Monte Carlo	$n_{L_{1}}$		$n_{L_{2}}$		$n_{L_{3}}$		$l_{L}$	
	R	p	R	p	R	p	R	p
$n_{L_{1}}$	-	-	-	-	-	-	-	-
$n_{L_{2}}$	0.851	0	-	-	-	-	-	-
$n_{L_{3}}$	0.839	0	1	0	-	-	-	-
$l_{L}$	0.369	0	0.269	0.007	0.269	0.008	-	-

**Table 7:** Comparison between experimental and Monte Carlo covariance matrices (3D: 20 µm; image: 0.1 pixel).

Experimental	$n_{L_{1}}$	$n_{L_{2}}$	$n_{L_{3}}$	$l_{L}$	Monte Carlo	$n_{L_{1}}$	$n_{L_{2}}$	$n_{L_{3}}$	$l_{L}$
$n_{L_{1}}$	1.21 E − 07	1.93 E − 07	1.74 E − 07	−3.69 E − 06	$n_{L_{1}}$	2.03 E − 07	1.31 E − 07	1.16 E − 07	5.96 E − 06
$n_{L_{2}}$	1.93 E − 07	3.16 E − 07	2.85 E − 07	−5.81 E − 06	$n_{L_{2}}$	1.31 E − 07	1.17 E − 07	1.05 E − 07	3.31 E − 06
$n_{L_{3}}$	1.74 E − 07	2.85 E − 07	2.57 E − 07	−5.26 E − 06	$n_{L_{3}}$	1.16 E − 07	1.05 E − 07	9.35 E − 08	2.89 E − 06
$l_{L}$	−3.69 E − 06	−5.81 E − 06	−5.26 E − 06	1.49 E − 06	$l_{L}$	5.96 E − 06	3.31 E − 06	2.89 E − 06	1.29 E − 06
